# Leptin receptor signaling via Janus kinase 2/Signal transducer and activator of transcription 3 impacts on ovarian cancer cell phenotypes

**DOI:** 10.18632/oncotarget.19873

**Published:** 2017-08-03

**Authors:** Janani Kumar, Hao Fang, Daniel R. McCulloch, Tamsyn Crowley, Alister C. Ward

**Affiliations:** ^1^ School of Medicine, Deakin University, Geelong, Victoria, Australia; ^2^ Centre for Molecular and Medical Research, Deakin University, Geelong, Victoria, Australia

**Keywords:** ovarian cancer, leptin receptor, JAK2, STAT3, cancer phenotypes

## Abstract

Ovarian cancer is a leading cause of cancer mortality in women world-wide. Considerable progress has been made to characterize the different subtypes of ovarian cancer, but specific therapies remain limited and prognosis poor. Cytokine signaling via the interleukin-6 receptor (IL-6R) family and related receptors has been implicated in a number of cancers, including those with an ovarian origin. The leptin receptor (LEPR) is structurally related to these receptors and utilizes similar downstream pathways. LEPR has diverse roles in metabolism, appetite and bone formation with obesity linked to both elevated levels of leptin and increased cancer incidence. This study investigated a potential role for LEPR signaling in ovarian cancer. Leptin stimulation led to increased proliferation, survival and migration of LEPR-expressing ovarian cancer cell lines, with the effects shown to be mediated by the downstream Janus kinase 2/Signal transducer and activator of transcription 3 (JAK2/STAT3) pathway. A significant correlation was identified between high co-expression of leptin and LEPR and decreased patient survival. This study collectively suggests that leptin/LEPR signaling via JAK2/STAT3 has the potential to significantly impact on pathogenesis in a subset of ovarian cancer patients who may benefit from strategies that dampen this pathway.

## INTRODUCTION

Ovarian cancer remains the most lethal gynecological cancer, and represents one of the major causes of cancer morbidity and mortality in women worldwide [1]. This is partly as a result of the largely asymptomatic nature of early disease, with women usually presenting with advanced disease when the tumor has already spread into the peritoneum and sometimes to distant metastatic sites [2]. In addition, there remains only limited knowledge of the molecular factors contributing to ovarian cancer etiology with which to inform specific therapeutic strategies. Despite treatment with aggressive surgery followed by chemotherapy, ovarian cancer patients experience <40% survival at 5 years, principally due to recurrent disease including metastasis [1].

Leptin is a cytokine with a wide variety of biological roles including regulation of energy metabolism, appetite, bone formation and angiogenesis [3]. High levels of leptin are observed in obese individuals [4], and these individuals have an increased risk of diabetes, fatty liver disease and cancer [5]. Indeed, leptin has been shown to have an important role in cell proliferation, invasion, metastasis and/or survival in several cancer types, such as breast, liver, colon, esophageal and endometrial cancers [6–10], including a role in ovarian cancer proliferation [11]. In breast cancer, the effects of leptin have been shown to be mediated through the activation of specific genes by STAT3, including cyclin D1 and c-Myc for proliferation [12] and VEGF and its receptor for angiogenesis [13].

Leptin exerts its action through a specific homodimeric receptor called leptin receptor (LEPR) [14]. LEPR is structurally related to the common signaling chain of the interleukin-6 receptor (IL-6R) family of cytokine receptors, glycoprotein 130 (GP130), as well as granulocyte colony-stimulating factor receptor (G-CSFR). Each of these receptor chains signals via the Janus kinase 2/Signal transducer and activator of transcription 3 (JAK2/STAT3) pathway [15]. Importantly, aberrant signaling by IL-6R and G-CSFR has been shown to contribute to key ovarian cancer phenotypes, including proliferation, migration and resistance against apoptosis [16–19].

These factors have led us to investigate the role of LEPR signaling in ovarian cancer. Analysis of ovarian cancer cell lines expressing functional LEPR revealed that leptin induced proliferation of adenocarcinoma cell lines and stimulated both survival and migration in both adenocarcinoma and teratocarcinoma cell types. These effects of leptin were shown to be mediated by the JAK2/STAT3 pathway. Further analysis revealed that high co-expression of leptin and LEPR correlated with reduced survival in ovarian cancer patients.

## RESULTS

### Expression of LEPR in ovarian cancer

Correlations between leptin levels and cancer have been described previously [5], while leptin signaling via LEPR has been implicated in breast and other cancers [8, 10]. Moreover, structurally-related members of the IL-6R family, including IL-6R and G-CSFR, have been shown to contribute to ovarian cancer progression [18, 19]. Therefore, the potential role of LEPR signaling in ovarian cancer biology was investigated. Preliminary experiments revealed vastly different levels of *LEPR* expression in a group of Grade 3 ovarian tumors (Supplementary Figure 1A) that was confirmed in a panel of 52 cell lines representing a range of different ovarian cell sub-types (Supplementary Figure 1B) and in 484 ovarian cancer samples (Supplementary Figure 2).

### Role of LEPR signaling in ovarian cancer cells

**Figure 1:**
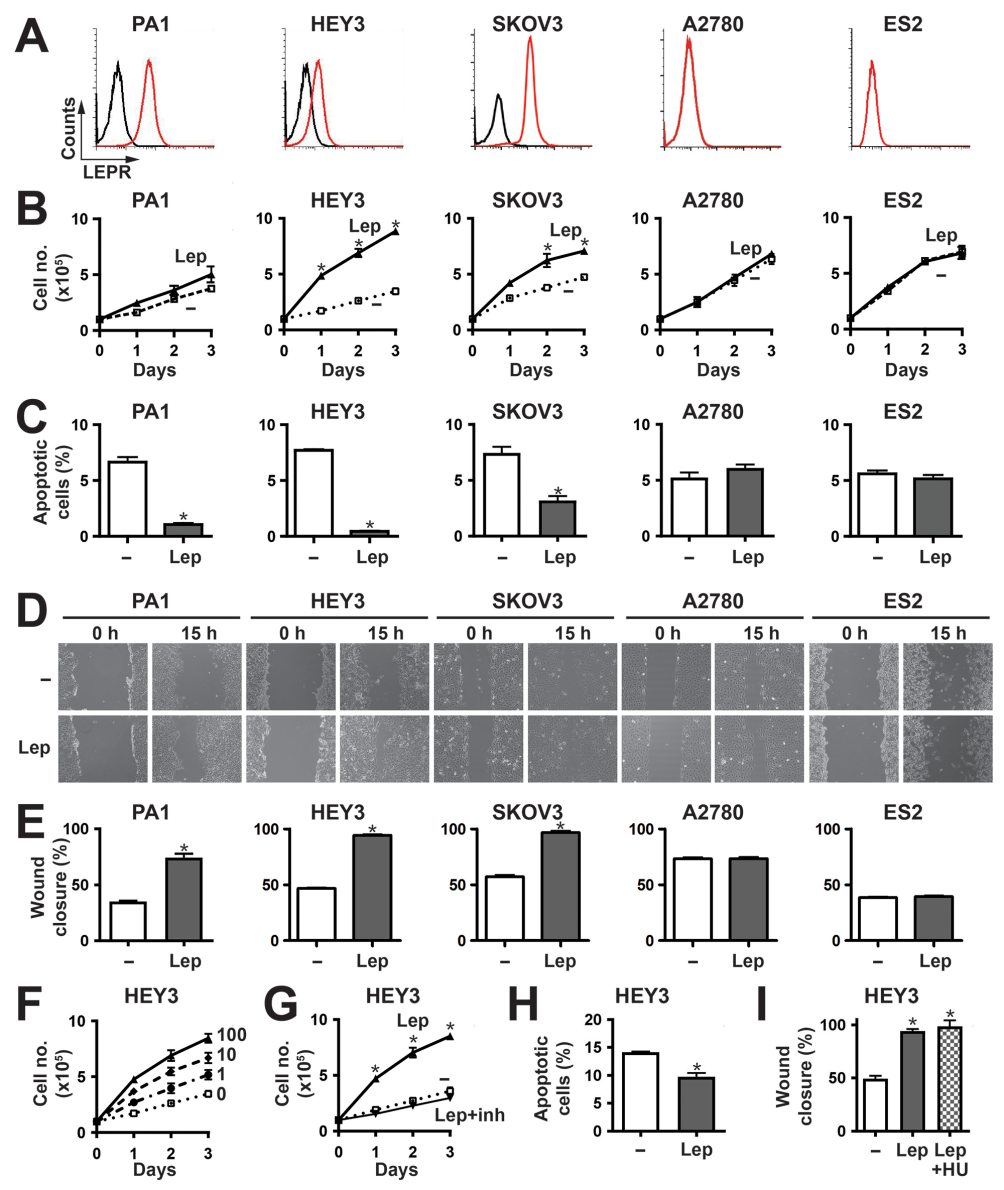
Effects of leptin/LEPR stimulation on ovarian cancer cell phenotypes **(A)** Expression of *LEPR* in a panel of representative ovarian cancer cell lines. The indicated cells were subjected to FACS analysis using anti-LEPR (red line) or an isotype control (black line) to quantify cell surface expression. **(B, F-G)** Proliferation assay. Cell counts for the indicated cell lines over a period of 3 days either untreated (–) or treated with 100 ng/ml leptin (Lep) (B,G) or a range of leptin concentrations (0, 1, 10 and 100 ng/ml) (F) alone, or in the presence of leptin inhibitor (inh) (G). The graphs represents the mean and SEM of three independent experiments (^*^p<0.05). **(C, H)** Apoptosis assay. Apoptosis of the indicated cell lines induced by either sodium azide (C) or cisplatin (H), when untreated (–) or treated with 100 ng/ml leptin (Lep). Graphs show the mean percentage and SEM of three independent experiments of apoptotic cells pre- and post-leptin treatment (^*^p<0.05). D-E,I. Migration assay. The indicated cell lines, either untreated (–) or treated with 100 ng/ml leptin (Lep) alone **(D-E)** or in combination with hydroxyurea (I), were subjected to wounding and imaged at 0 h and 15 h. Representative images are provided (D), along with graphs showing the mean wound closure and SEM from three independent experiments **(E,I)** (^*^p<0.05).

To identify suitable cell models to investigate the functional significance of LEPR expression, FACS analysis was performed on a set of ovarian cancer lines in-hand. This identified adenocarcinoma (HEY3, SKOV3) and teratocarcinoma (PA1) cell lines expressing LEPR, along with adenocarcinoma (A2780) and clear cell carcinoma (ES2) cell lines that did not express detectable LEPR (Figure 1A). RT-PCR analysis confirmed the expression of the ‘long’ LEPR isoform in HEY3, SKOV3 and PA1 cells (Supplementary Figure 3), and data not shown which is the form capable of intracellular signaling [14, 20, 21], with leptin treatment able to increase this isoform in HEY3 cells only (Supplementary Figure 3).

Cytokines are known to stimulate proliferation, survival and migration in responsive cells, and so these parameters were assessed in response to leptin stimulation. Leptin elicited a significant increase in proliferation in LEPR-positive HEY3 and SKOV3 cells, a marginal but non-significant response in PA1 cells, and no response in the LEPR-negative A2780 and ES2 cells (Figure 1B). This response was dose-dependent (Figure 1F) and could be inhibited by a specific leptin inhibitor (Figure 1G). With respect to survival, the cell lines showed very low levels of apoptosis even in low serum, and so apoptosis was induced by the addition of sodium azide in order to evaluate potential anti-apoptotic responses. Leptin provided variable but statistically significant protection against apoptosis in the LEPR-positive PA1, HEY3 and SKOV3, but not in A2780 and ES2 cells (Figure 1C). This effect was confirmed in HEY3 cells treated with the clinically-relevant therapeutic agent cisplatin (Figure 1H). Cell migration was also examined in these cells using a wound-healing assay, and was found to be increased upon leptin stimulation in the cell lines expressing LEPR (PA1, HEY3 and SKOV3) but not in the LEPR-negative (A2780 and ES2) cells (Figure 1D, 1E). This effect was similar in the presence of the proliferation inhibitor hydroxyurea (Figure 1I), indicating it was independent of cell division.

### LEPR acts via the JAK2/STAT3 pathway in ovarian cancer cells

**Figure 2:**
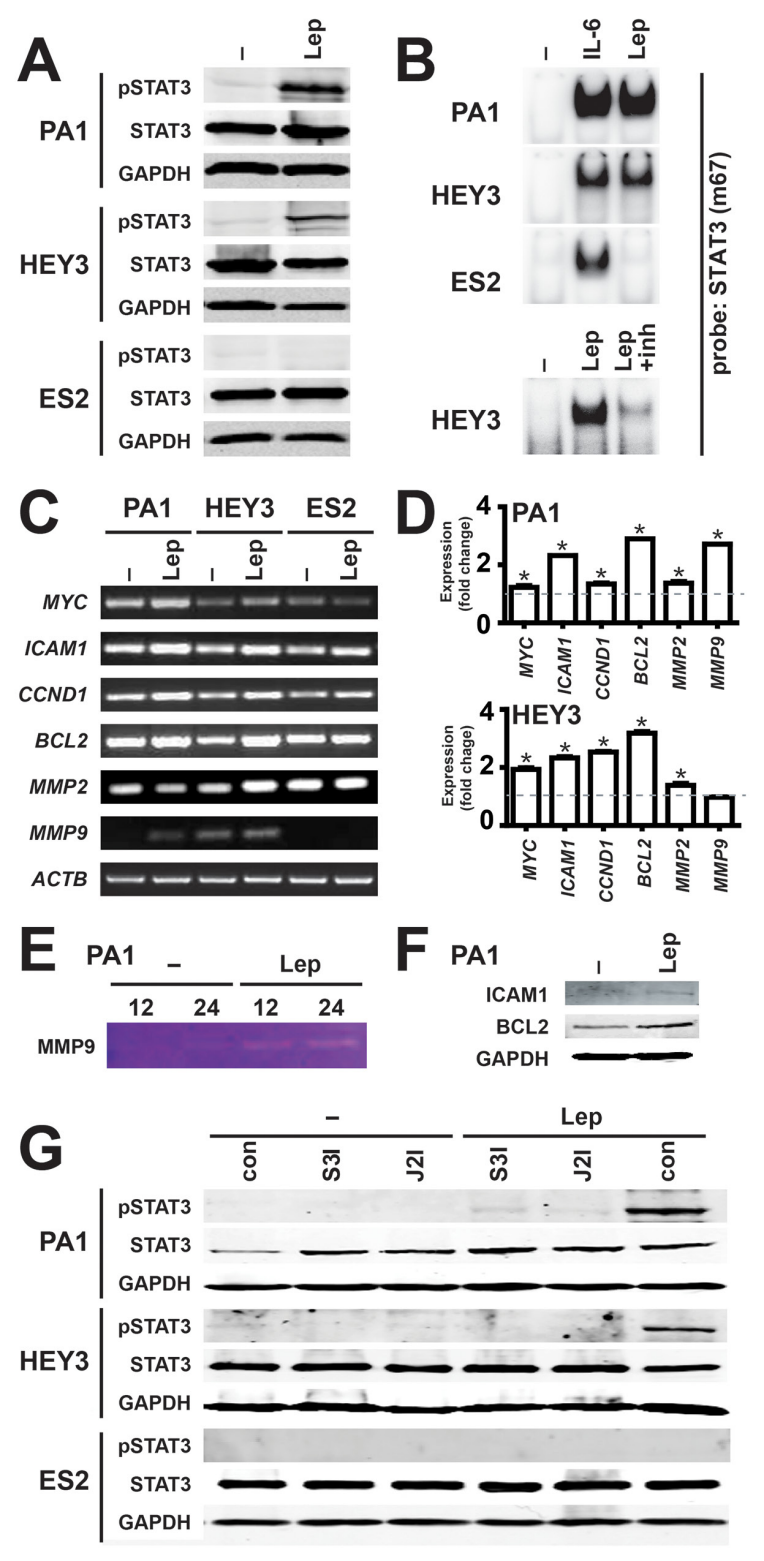
Activation of the JAK2/STAT3 pathway by leptin/LEPR in ovarian cancer **(A)** Activation of STAT3 by leptin. Cell lysates from the indicated cell lines, either untreated (–) or treated for 45 min with 100 ng/ml leptin (Lep), were analyzed for STAT3 activation by Western blot with anti-phospho-STAT3, along with anti-STAT3 and anti-GAPDH to confirm equivalent loading. Experiments were performed thrice and blots are representative from one experiment (Supplementary Figure 4). **(B)** Induction of STAT3 DNA binding activity by leptin. Nuclear extracts from the indicated cell lines, either untreated (–) or treated for 45 min with 100 ng/ml leptin (Lep), interleukin-6 (IL-6), or leptin and leptin-inhibitor (inh) and analyzed for STAT3 activation by EMSA with a STAT3 (m63) probe. Experiments were performed thrice and blots are representative of one experiment. **(C-D)** Expression of STAT3 responsive genes by leptin. Cells, either untreated (–) or treated with 100 ng/ml leptin (Lep), were analyzed by semi-quantitative RT-PCR (C), with the fold change and SEM quantified by subsequent Real-Time RT-PCR (D) (^*^p<0.05). **(E-F)** Induction of STAT3 target gene products by leptin. PA1 cells, either untreated (–) or treated with 100 ng/ml leptin (Lep), were analyzed by gelatin zymography for MMP9 (E) and Western blot analysis for ICAM-1, BCL2 and GAPDH (F). **(G)** Leptin-induced STAT3 activation is blocked by specific inhibitors of the JAK2/STAT3 pathway. Lysates were prepared from cells, either untreated (–) or treated with 100 ng/ml leptin (Lep), without inhibitors (con) or with specific inhibitors for STAT3 (S3I) or JAK2 (J2I), as indicated, and analyzed for STAT3 phosphorylation as per panel A (Supplementary Figure 5).

Leptin is known to activate a number of different signaling pathways, particularly the JAK2/STAT3 pathway [21], including in cancer cells [6]. To investigate whether the LEPR utilizes this pathway in ovarian cancer cells, representative cell lines were analyzed for activation of STAT3. Leptin stimulation led to a robust increase in phosphorylated STAT3 (pSTAT3) that correlated with the LEPR expression levels in PA1 and HEY3, but this was not observed in LEPR-negative ES2 cells, despite comparable levels of total STAT3 and GAPDH used to confirm equivalent loading (Figure 2A). To determine whether the effects on STAT3 phosphorylation translated to active nuclear STAT3, nuclear extracts were analyzed by EMSA using a STAT3-binding site. Leptin induced STAT3 binding that mirrored pSTAT3 levels, with strong leptin-dependent binding in PA1 and HEY3 cells, but not in ES2 cells, despite robust STAT3 activation by IL-6 in each cell line (Figure 2B). The expression of representative STAT3 responsive genes following leptin stimulation was examined using semi-quantitative RT-PCR: *ICAM1*, *MMP2* and *MMP9* for migration, *MYC* and *CCND1* for proliferation, and *BCL2* for survival. Leptin treatment increased the expression of each of these genes in the LEPR-positive cell lines (PA1 and HEY), but produced no change in the LEPR-negative cell line (ES2), with *ACTB* used as a control (Figure 2C). The increases in gene expression were subsequently quantified by Real-Time RT-PCR, which confirmed statistically significant induction in all but one case (Figure 2D). Induction of the encoded protein was also verified in PA1 cells by zymography for MMP9 (Figure 2E) and by Western blot analysis for ICAM1 and BCL2 (Figure 2F). Importantly, specific inhibitors for both JAK2 and STAT3 effectively blocked leptin-induced STAT3 activation (Figure 2G) and gene induction (data not shown) in PA1 and HEY3 cell lines, confirming a functional JAK2-STAT3 pathway. Similar results were obtained in SKOV3 cells (data not shown).

**Figure 3:**
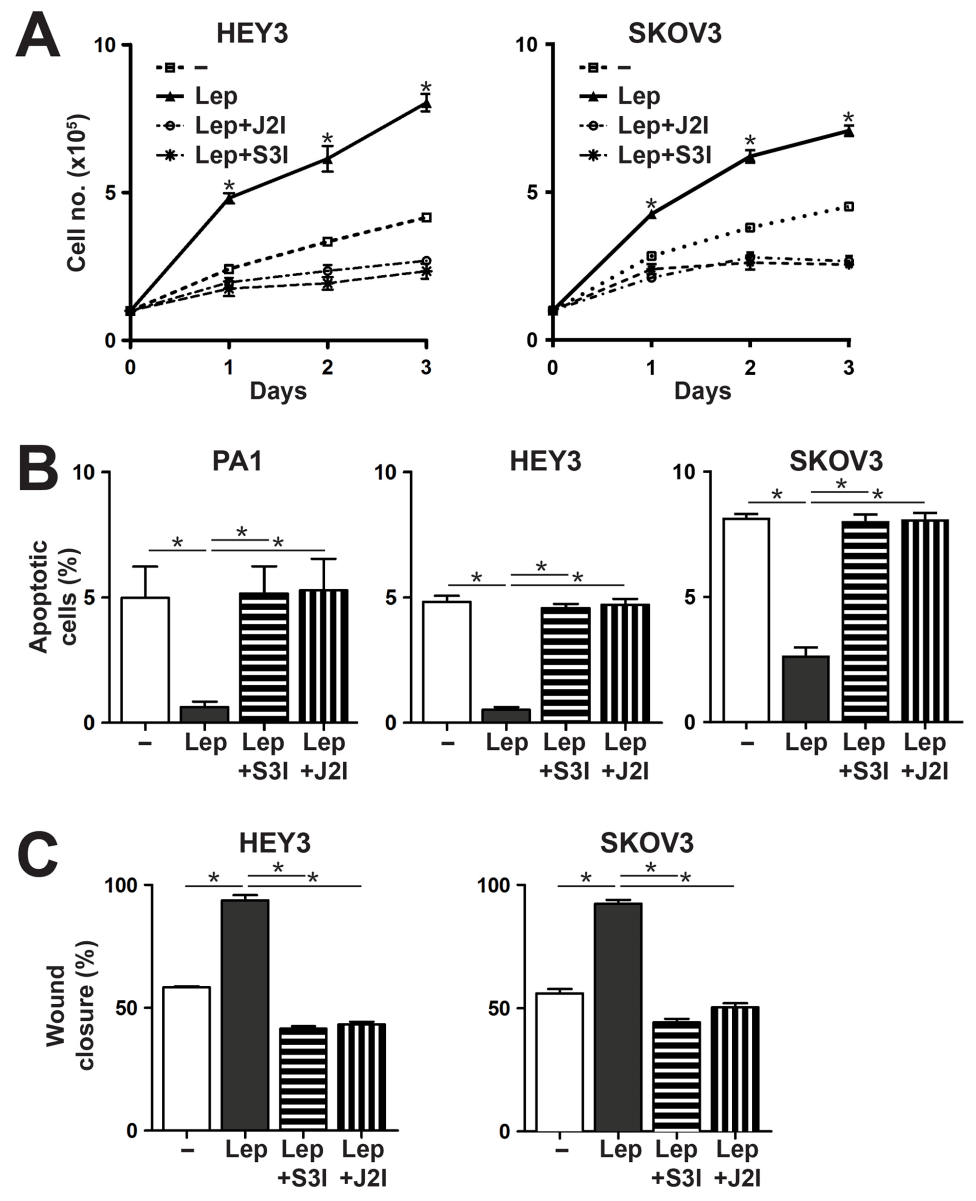
Effects of Leptin/LEPR on cell proliferation, survival and migration are mediated via the JAK2/STAT3 pathway **(A)** Proliferation assay. Cell counts over a period of 3 days for the cells indicated either untreated (–) or treated with 100 ng/ml leptin (Lep), with or without specific inhibitors for STAT3 (S3I) or JAK2 (J2I), as indicated. The graph represents the mean and SEM of three independent experiments (^*^p<0.05). **(B)** Apoptosis assay. Percentage of apoptosis for the indicated cell lines when treated with sodium azide alone, or with leptin (Lep) treatment, in the presence or absence of inhibitors for STAT3 (S3I) and JAK2 (J2I). The graph shows mean and SEM (n=3; ^*^p<0.05 compared to untreated; ^**^p<0.05 compared to leptin treated). **(C)** Migration assay. Percentage of wound closure for the indicated cell lines, either untreated (–) or treated with 100 ng/ml leptin (Lep), in the presence or absence of inhibitors for STAT3 (S3I) or JAK2 (J2I), as indicated, showing mean and SEM (^*^p<0.05).

To confirm that signaling via the JAK2-STAT3 pathway also contributed to the leptin/LEPR-induced phenotypes, responsive cell lines were re-analyzed for these phenotypes in combination with the specific inhibitors for JAK2 and STAT3. Both inhibitors were able to suppress leptin-mediated proliferation (Figure 3A), survival (Figure 3B) and migration (Figure 3C) in LEPR-expressing ovarian cancer cells.

### High leptin/LEPR coexpression represents a poor prognostic factor in ovarian cancer

**Figure 4:**
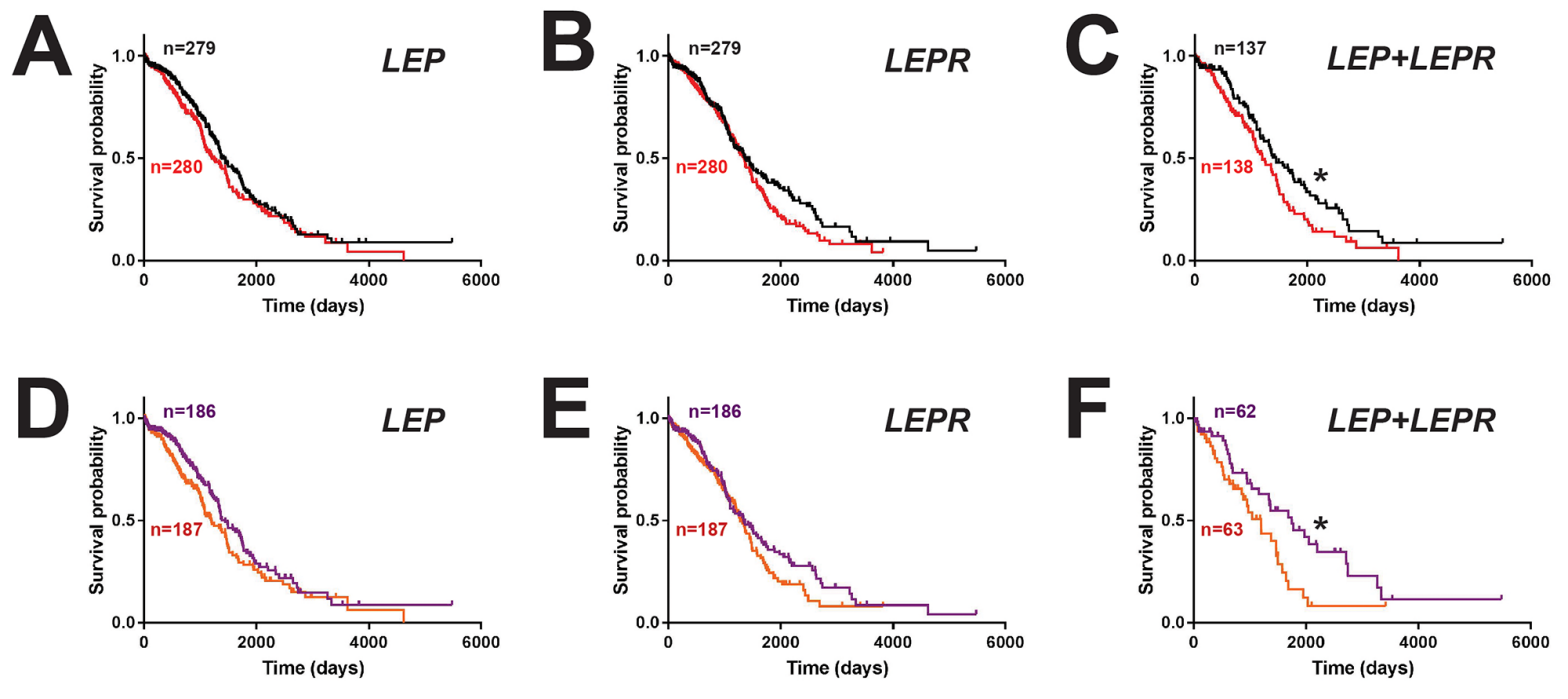
Impact of leptin/LEPR expression on ovarian cancer patient survival Overall survival curves of ovarian cancer patients expressing high (above median, red) versus low (below median, black) levels of *LEP* **(A)**, *LEPR* **(B)** or both *LEP* and *LEPR* **(C)**, or those expressing higher (top tertile, orange) versus lower (bottom tertile, purple) levels of *LEP* **(D)**, *LEPR* **(E)** or both *LEP* and *LEPR* **(F)** (^*^p<0.05).

To assess the clinical consequences of *LEP* and *LEPR* expression, survival analysis was performed on ovarian cancer patients, comparing groups expressing *LEP* or *LEPR* above and below the median level. This revealed a non-significant trend for decreased survival in patients with high levels of either *LEP* (hazard ratio 1.20, 95% CI 0.95-1.51) (Figure 4A) or *LEPR* (hazard ratio 1.23, 95% CI 0.97-1.55) (Figure 4B). However, a statistically significant decrease in survival was identified for patients with high expression of both *LEP* and *LEPR* (hazard ratio 1.45, 95% CI 1.05-1.99; median survival 1204 v. 1448 days) (Figure 4C), despite no difference in expression of JAK2 or STAT3 (Supplementary Figure 7). This effect was heightened in patients in the top tertile for *LEP* (hazard ratio 1.27, 95% CI 0.96-1.68) (Figure 4D) or *LEPR* (hazard ratio 1.24, 95% CI 0.93-1.64) (Figure 4E) and particularly for both *LEP* and *LEPR* compared to those in the bottom tertiles (hazard ratio 1.77, 95% CI 1.07-2.92; median survival 1199 v. 1746 days) (Figure 4F).

## DISCUSSION

Leptin signaling via LEPR has a wide variety of roles in regulating energy metabolism, appetite regulation, bone formation and angiogenesis [3, 22, 23]. LEPR achieves this by initiating downstream signal cascades, including the JAK2/STAT3 pathway. Several studies have indicated that LEPR signaling can induce cancer cell adhesion, angiogenesis, proliferation, survival, migration and stem cell-ness in several malignancies [8, 10, 24-27]. However, whether LEPR-mediated signaling impacted on ovarian cancer remained poorly understood.

This study identified leptin-induced migration of LEPR-positive ovarian cancer cells mediated via JAK2/STAT3. Previous studies have shown that leptin can induce cancer cell migration via the MAP kinase in prostate cancer [26], via the AKT pathway in endothelial cells [28], via the NF-kB pathway in glioma cells [29], and via the JAK/STAT, PI3K/AKT, and MAPK pathways in hepatocellular carcinoma cells [8]. This indicates that leptin can utilize multiple pathways to influence cancer cell migration, although we showed that in ovarian cancer cells inhibition of the JAK2/STAT3 pathway was sufficient to block this effect. A role for leptin/LEPR in the survival of ovarian cancer cells was also demonstrated, similarly through the JAK2/STAT3 pathway, consistent with a previous study showing an alternate JAK2 inhibitor was able to induce apoptosis in ovarian cancer cells [30].

Our data has also shown that leptin/LEPR signaling enhanced proliferation in the HEY3 and SKOV3 adenocarcinoma cell lines also via JAK2/STAT3. Two previous studies have demonstrated leptin-mediated proliferation of ovarian cancer cells via the MAPK pathway and PI3K pathways [11, 31]. Importantly, both pathways typically lies downstream of JAK2 and so are likely blocked with the JAK2 inhibitors used in this study. However, since leptin-induced proliferation was also abrogated with the STAT3 inhibitor, it appears that STAT3-mediated pathways contribute to proliferation independently of MAPK or PI3K. Leptin has also been reported to activate the estrogen receptor pathway [32], providing an alternative mechanism to impact on proliferation. Collectively, this work has shown that LEPR signaling is able to positively impact on several key tumor-promoting phenotypes that are also influenced by other IL-6R-related receptors, such as IL-6R itself [19] and G-CSFR [18].

Amongst the LEPR-positive cell lines proliferation, migration and survival responses did not correlate well with relative LEPR expression, although this did correlate with the extent of leptin-mediated STAT3 activation. This is complicated by the fact that leptin induced LEPR expression in HEY3 cells (Supplementary Figure 3). Moreover, use of a LEPR-Fc ‘decoy’ abrogated leptin responses ruling out the possibility that some effects were LEPR-independent. Importantly, expression of key STAT3-target genes (eg. *MYC*) correlated well with the relevant phenotypes observed.

Obesity is considered an important etiological factor for cancer that is associated with increased incidence of many common types of cancers [33]. Elevated levels of leptin are observed in obese individuals [4], with several studies reporting a correlation between obese individuals, high leptin levels and enhanced risk of breast, prostate and endometrial cancers [26, 34–36]. We identified that high tumor expression of *LEP* and *LEPR* separately showed a trend toward poor survival. This was supported by another study for *LEP* [37] and our own analysis of an alternate data set [38] for *LEPR* that emphasised a role in long-term survival (hazard ratio 1.18, CR 1.03-1.34) (Supplementary Figure 6). Furthermore, high co-expression of *LEP* and *LEPR* within the tumor clearly represented a poor prognostic factor for epithelial ovarian cancer, consistent with another recent study [39]. However, the major source of leptin production is subcutaneous fat [40]. Thus, individuals with LEPR-positive cancers could be particularly susceptible to high leptin levels seen in obesity irrespective of *LEP* expression in the tumor itself. High BMI has been associated with increased risk of ovarian cancer [41, 42], although not in high-grade serous cancers [41]. However, amongst women with ovarian carcinoma, increased BMI was associated with reduced disease-specific survival [43–45], including in high-grade serous cancer [45]. Importantly, amongst women with epithelial ovarian cancer, there was a significant correlation between BMI and leptin levels, with patients with a low leptin:adiponectin ratio demonstrating statistically longer disease-specific survival [46]. This suggests weight control could potentially contribute to positive outcomes in ovarian cancer, and particularly those patients with LEPR-positive tumors. Additional therapeutic options should also be considered for these patients, such as JAK/STAT inhibitors, which are already being used in various clinical settings [47].

This paper has demonstrated that ovarian cancer cells expressing LEPR can signal through the JAK2/STAT3 pathway to contribute to key cancer phenotypes. This was consistent with high co-expression of *LEP* and *LEPR* correlating with poor survival of ovarian cancer patients. This suggests that further research into weight control strategies and JAK/STAT inhibitors in this clinical setting are warranted.

## MATERIALS AND METHODS

### Cell lines and clinical samples

The human epithelial ovarian cancer line HEY3 was obtained from the Royal Women’s Hospital Melbourne, A2780 from Deakin University, while PA1, SKOV3, and ES2 were obtained from American Type Culture Collection (Rockville, MD). Cell lines were grown as monolayers in 25 or 75 cm^2^ flasks (BD, Australia) in complete growth medium consisting of Basal Medium Eagle for PAI, McCoy’s 5a for SKOV3 and ES2, Dulbecco’s Modified Eagle’s Medium (DMEM) HEY3, and RPMI1640 for A2780, in each case supplemented with 10% (v/v) heat-inactivated fetal bovine serum and 2 mM glutamine (Invitrogen Corporation, Melbourne, Australia) in the presence of 37°C with 5% CO_2_. Recombinant human leptin (Sigma Aldrich, Melbourne, Australia) was used at 0-100 ng/ml, along with recombinant mouse LEPR-Fc chimera (Leptin inhibitor, 0.5 μg/ml) (R&D Systems, Minneapolis, MN, USA), WP1066 (JAK2 inhibitor, 2.5 μM) and LLL12 (STAT3 inhibitor, 2.5 μM) (BioVision, Mountain View, CA, USA), cisplatin (20 μg/ml) (Pfizer, Perth, WA, Australia), sodium azide (0.5%) and hydroxyurea (50 μM) (Sigma Aldrich), as required. Grade 3 serous ovarian carcinomas were collected from patients after obtaining written informed consent under protocols approved via the Victorian Cancer Biobank and approved by the Deakin University Human Research Ethics Committee (DUHREC#2010-104). All patients had undergone chemotherapy with standard agents.

### Receptor expression analysis

Confluent cell lines were harvested for fluorescence activated cell sorter (FACS) analysis. Cell monolayers were washed twice with phosphate buffered saline (PBS), detached with 0.25% (w/v) trypsin-EDTA solution, collected by centrifugation and washed a further two times with PBS. Approximately 1×10^6^ cells were fixed using 4% (w/v) paraformaldehyde (PFA) and subsequently blocked for 30 min in PBS containing 10% (v/v) goat serum. After incubation, the cells were washed twice with PBS, incubated with 1 μg/μl anti-LEPR antibody or isotype control (AbCam, USA) at room temperature, then washed three times in PBS before incubation with goat anti-rabbit Alex Fluro® 488 (Invitrogen) at 1/500 dilution for 60 min at room temperature. Samples were analysed on a FACS Canto (BD Bioscience) using FACS DIVA and FlowJo software, in comparison to appropriate controls.

### Growth assay

Growth rates were assessed as previously described [19]. Briefly, 1×10^5^ cells were seeded onto a six well plate in appropriate media. Each day the cells were trypsinized, resuspended in medium and counted using a hemocytometer. Each experiment was performed in triplicate for each harvest and repeated thrice.

### Apoptosis assay

Apoptosis was quantified by double-staining with Annexin V-FITC and propidium iodide (PI) using an Apoptosis Detection Kit (BD Biosciences), as we have described previously [48].

### Cell migration assay

The migratory potential of ovarian cancer cells was assessed using a wound healing assay, as previously described [19]. Briefly, cells were grown to a confluent monolayer in a 6-well plate and ‘wounded’ with a sterile 200 μl pipette tip. Three representative fields were marked and imaged at 0 h and 15 h. Images were captured using an Olympus SC20 camera, with the width of the scratches was measured at each time point and quantified using Cell Profilier®.

### Western blot analysis

Proteins were extracted using RIPA Lysis buffer containing Phosphatase Inhibitor Cocktail (Sigma Aldrich, Sydney, Australia) according to the manufacturer’s instructions. Protein concentration was quantified using a BCA Protein Determination Kit (Pierce) as per the manufacturer’s instructions and absorbance read at 562 nm on a PerkinElmer VICTOR X Multilabel Plate Reader. Western blots were performed as previously described [19], utilizing rabbit antibodies against phospho-STAT3 (Tyr 705), total-STAT3, BCL2 and ICAM-1 (Cell Signaling, Beverly, MA, USA), and a mouse monoclonal antibody against GAPDH (Millipore Technologies, Sydney, Australia) at 1:1000 dilution, followed by anti-rabbit IRDye™ 680 CW and anti-mouse IRDye™ 800 CW (LI-COR Biosciences, Nebraska, USA) at 1:10000 dilution in 5% (w/v) BSA in TBS containing 0.1% Tween. Images were obtained using an Odyssey Imaging System (LI-COR Biosciences) and analysed with ImageJ (NIH).

### Gelatin zymography

Conditioned media from cells cultured for 12-24 h was mixed with sample buffer and subjected to SDS-PAGE using gels containing 10% (w/v) gelatin. These were soaked three times for 30 min at room temperature in PBS containing 2.5% Triton X-100, before incubation for 24 h at 37°C and then staining with 0.1% Coomassie Brilliant Blue R-250.

### EMSA

Nuclear extracts were prepared and analysed by electrophoretic mobility shift assay (EMSA) using the m67 high affinity STAT binding site probe, as described previously [48].

### Reverse transcription-polymerase chain reaction

RNA extraction and reverse transcription-polymerase chain reaction (RT-PCR) were performed as described [19] using the following primer pairs: *LEPR* long form 5’-TTGTGCCAGTAATTATTTCCTCTT and 5’-ATAGCTTTTTCATTCTTTGGTGTG, *LEPR* short form 1 reverse 5’-CTGTGGCCTTCCGCAGTG, *LEPR* short form 2 reverse 5’-ACCTCCACCCAGTAGTTCCTT, *LEPR* short form 3 reverse 5’-AGTTGGCACATTGGGTTCAT, *LEP* 5’-GAAGACCACATCCACACACG and 5’-AGCTCAGCCAGACCCATCTA, *LEP* 5’-GAAGACCACATCCACACACG and 5’-AGCTCAGCCAGACCCATCTA, *ICAM1* 5’-GGCTGGAGCTGTTTGAGAAC and 5’-ACTGTGGGGTTCAACCTCTG, *CCND1* 5’-CCTAAGTTCGGTTCCGATGA and 5’-ACGTCAGCCTCCACACTCTT, *BCL2* 5’-GGATGCCTTTGTGGAACTGT and 5’-AGCCTGCAGCTTTGTTTCAT, *MMP2* 5’-ATGACAGCTGCACCACTGAG and 5’-ATTTGTTGCCCAGGAAAGTG, *MMP9* 5’-TTGACAGCGACAAGAAGTGG and 5’-GCCATTCACGTCGTCCTTAT, *MYC* 5’-TACCCTCTAACGACAGCAG and 5’-TCTTGACATTCTCCTCGGTG, and *ACTB* 5’-GGACTTCGAGCAAGAGATGG and 5’-AGCACTGTGTTGGCGTACAG. PCR products were visualized on 1-3% (w/v) agarose gels containing SYBR® Safe (Invitrogen) and imaged using a Chemidoc XRS Molecular Imager System (Bio-Rad). Gene expression was measured by quantitative RT-PCR (qRT-PCR) on an Agilent Stratagene MX3000P. Reactions (25 μL) contained 3.125 μL nuclease-free water, 3.125 μL cDNA template (1:10 dilution), 12.5 μL iQ SYBR Green Supermix (Bio-Rad) and 3.125 μL each of forward and reverse primer (2.4 μM). Typical PCR conditions consisted of 95°C for 30 sec, followed by 45 cycles of 95°C for 10 sec, 60°C for 30 sec, 72°C for 20 sec, then 95°C for 1 min, 58°C for 1 min, and finally incremental increases (0.5°C) from 58°C to 95°C to establish the melting curve for each sample. Appropriate control reactions were performed to ensure products were not the result of DNA contamination or due to primer-dimer formation. Data retrieved from these assays were analysed using the Livak method [49].

### Bioinformatic analysis

Expression data for 52 ovarian cancer cell lines of diverse subtypes were extracted from the Cancer Cell Line Encyclopedia (CCLE) (http://www.broadinstitute.org/ccle/home), which uses the Affymetrix U133+2 array platform (downloaded 18 Sep 2014). Expression data for 586 primary serous adenocarcinomas of the ovary and 8 normal ovary samples were obtained from The Cancer Genome Atlas (TCGA) (http://cancergenome.nih.gov), which uses the Affymetrix U133A array platform, along with clinical details for the 559 ovarian cancer patients with available survival data (Table 1) (downloaded 6 Mar 2014). Expression data were converted to a single value for each probe set using Robust Multi-array Average (RMA) approach with quartile normalization [50]. Survival curves were generated from this database, as well as one of approximately 1300 ovarian cancer patients with predominantly serous carcinoma (accessed 30 Nov 2015) [38], with comparisons based on relative *LEP* and *LEPR* expression using the Kaplan Meier method and log-rank hazard model.

**Table 1:** Clinical characteristics of patients in the TCGA ovarian cancer cohort

Clinical parameter	Details
**Total patient cohort**	559
**Age at time of pathology (n=549)**	
**- Mean:**	59.7 ± 11.6 years
**- Range:**	26-89 years
**Cancer stage (n=546)**	
**- I:**	13 (IA=1, 1B=3, IC=9)
**- II:**	27 (IIA=3, IIB=4, IIC=20)
**- III:**	424 (IIIA=7, IIIB=23, IIIC=394)
**- IV:**	82
**Radiation therapy (n=559)**	
**- yes**	32
**Chemotherapy (n=541)**	
**- yes**	529
**- Carboplatin**	486
**- Paclitaxel**	485
**- Cisplatin**	156
**- Doxorubicin**	154
**- Gemcitabine**	144
**- Topotecan**	122
**- Doxetaxel**	117

### Statistical analysis

The cell phenotype parameters investigated follow a normal distribution and were analysed by a 2-sample Student’s *t* test for pairwise comparisons of independent samples and One-way analysis of variance (ANOVA) for multiple comparisons using GraphPad Prism and SPSS software packages. Patient survival data was analyzed with Mantel-Cox methodology in GraphPad Prism. In each case, *p*<0.05 was considered statistically significant.

## SUPPLEMENTARY MATERIALS FIGURES


