# Supplementary material for: Leptin receptor signaling via Janus kinase 2/Signal transducer and activator of transcription 3 impacts on ovarian cancer cell phenotypes
Source: Oncotarget. 2017 Aug 3;8(55):93530–40. doi: 10.18632/oncotarget.19873 (PMC5706816; doi:10.18632/oncotarget.19873)
Supplement: Supplementary file 1 [file oncotarget-08-93530-s001.pdf]

Leptin receptor signaling via Janus kinase 2/Signal transducer and activator of transcription 3 impacts on ovarian cancer cell phenotypes

SUPPLEMENTARY MATERIALS

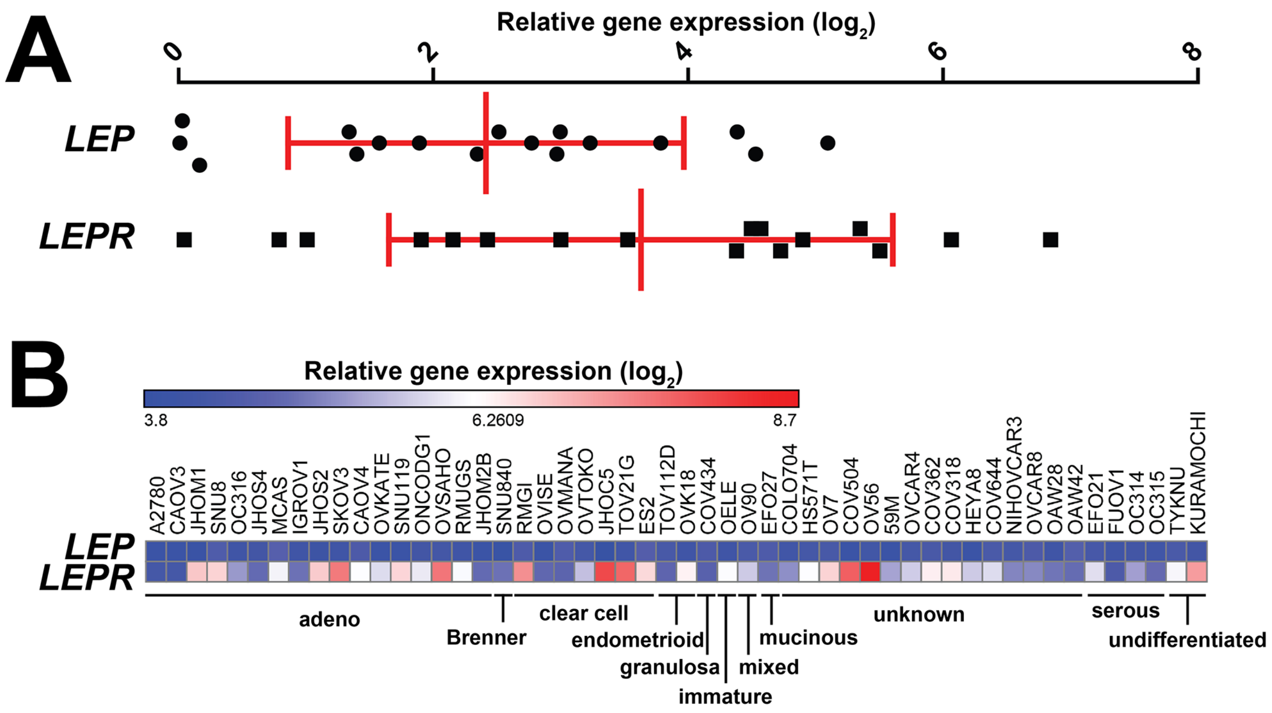

Supplementary Figure 1: Expression of leptin/LEPR in ovarian cancer. (A) Relative gene expression of *LEP* and *LEPR* in Grade 3 ovarian carcinoma samples was analyzed by qRT-PCR and normalized relative to *ACTB*, as indicated, showing mean and SEM. **(B)** Relative gene expression of *LEP* and *LEPR* in 52 ovarian cancer cell lines, which are shown along with their subtype designation. This is presented as a 'heat map' with the range of expression levels indicated in the key.

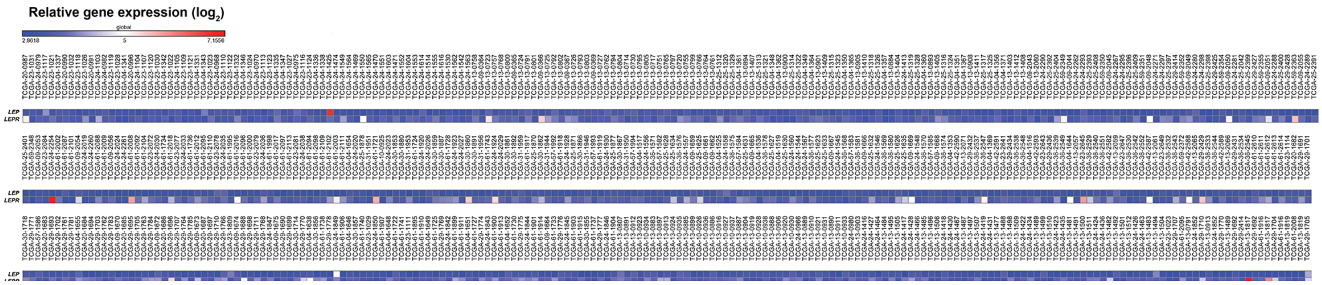

Supplementary Figure 2: Expression of leptin/LEPR in ovarian cancer patients. Relative gene expression of *LEP* and *LEPR* in 484 ovarian cancer samples, presented as a 'heat map' with the range of expression levels indicated in the key.

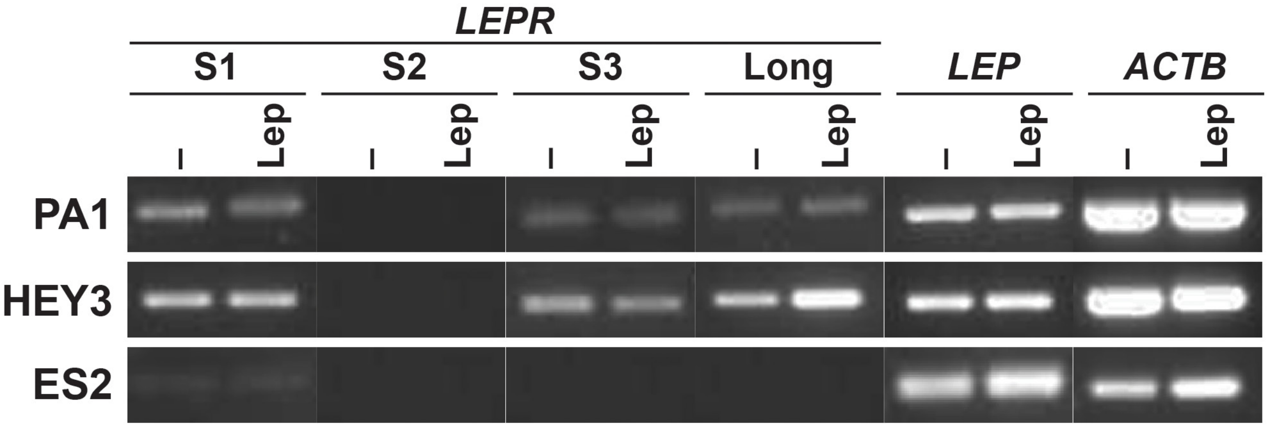

Supplementary Figure 3: Expression of leptin and LEPR isoforms in ovarian cancer cells. Expression of *LEP* and *LEPR* in ovarian cancer cell lines. Cells, either untreated (–) or treated with 100 ng/ml leptin (Lep), were analysed by RT-PCR for expression of the indicated gene and isoform, including *ACTB* as a control.

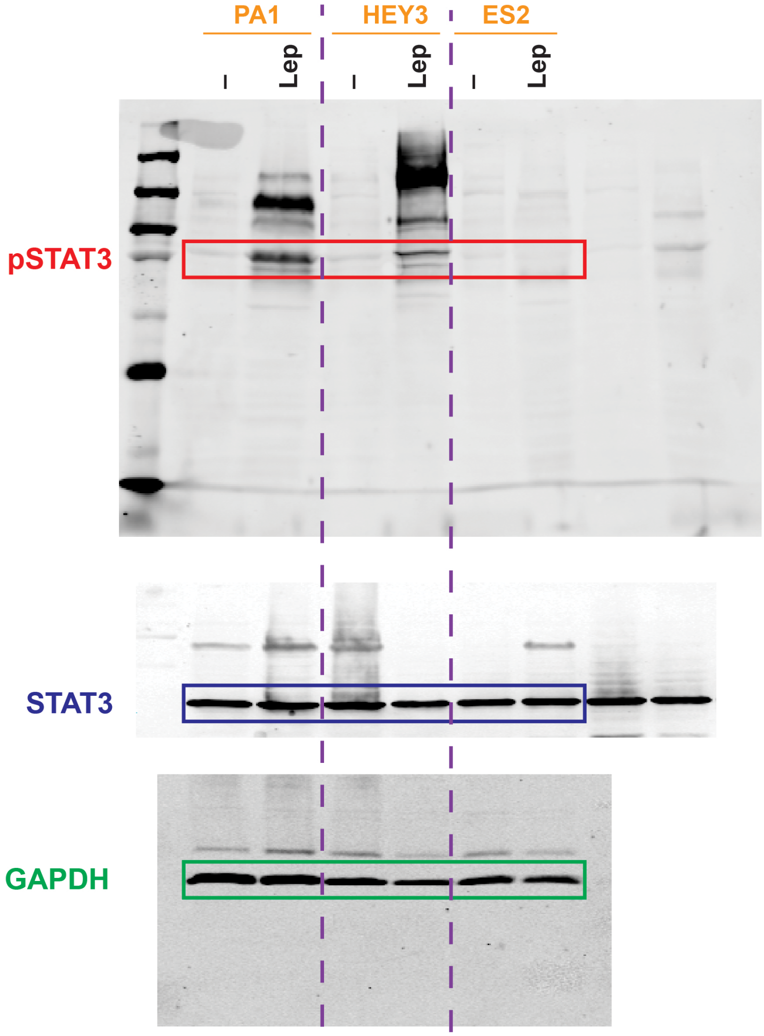

Supplementary Figure 4: STAT3 activation by leptin. Lysates from the indicated cell lines, either untreated (–) or treated for 45 min with 100 ng/ml leptin (Lep), were analyzed Western blot with anti-phospho-STAT3 (upper), along with anti-STAT3 (middle) and anti-GAPDH (lower) to confirm equivalent loading. Shown is a representative of three independent experiments.

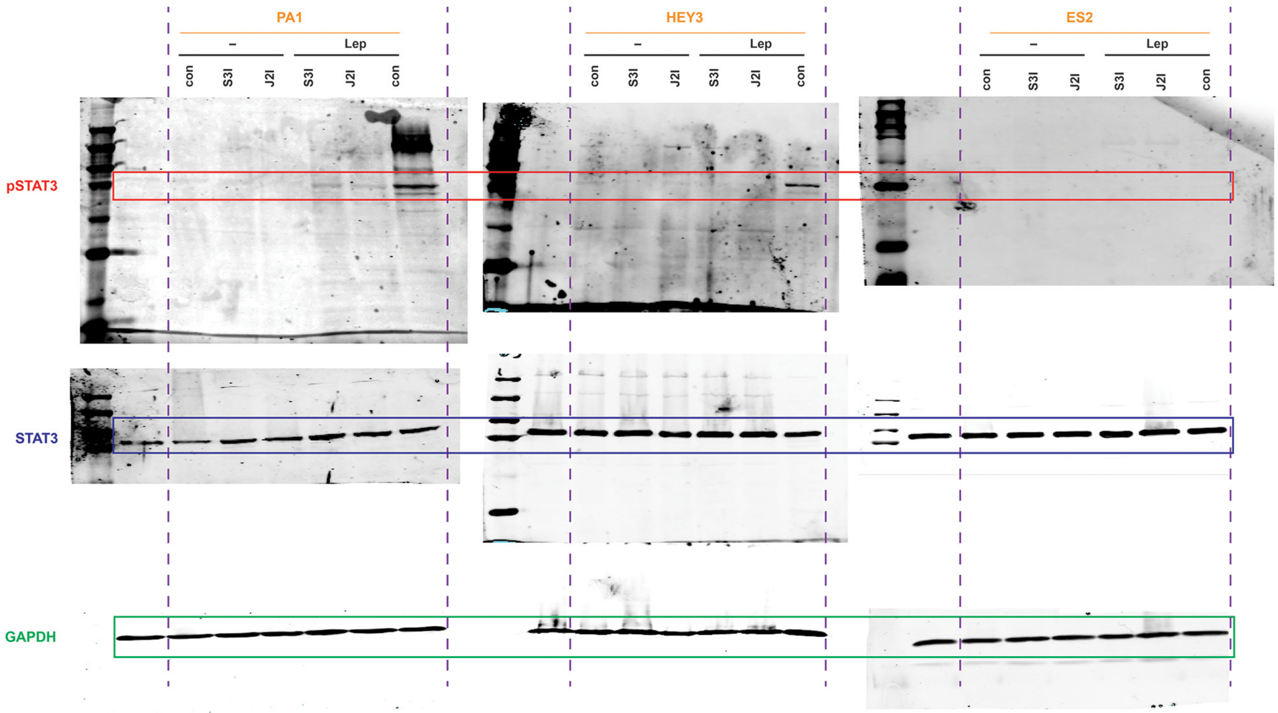

Supplementary Figure 5: Inhibition of leptin-induced STAT3 activation by JAK2 and STAT3 inhibitors. Lysates were prepared from cells, either untreated (–) or treated with 100 ng/ml leptin (Lep), without inhibitors (con) or with specific inhibitors for STAT3 (S3I) or JAK2 (J2I), as indicated, and analyzed for STAT3 activation as described in Supplementary Figure 4. Shown is a representative of three independent experiments.

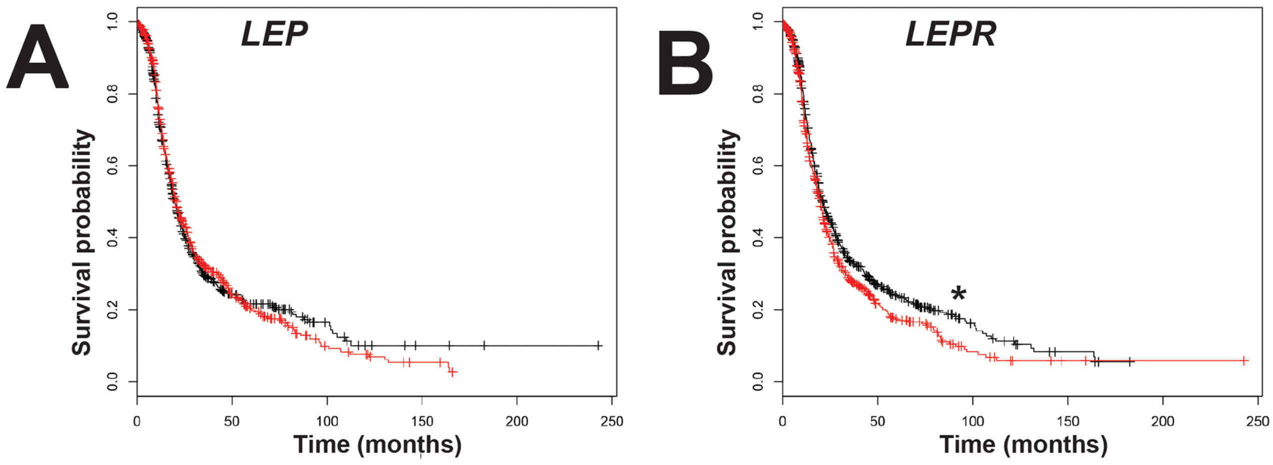

Supplementary Figure 6: Impact of leptin/LEPR expression on ovarian cancer patient survival. Overall survival curves of serous ovarian cancer patients expressing high (above median, red) versus low (below median, black) levels of *LEP* **(A)** or *LEPR* **(B)** ($^{*}p$<0.05).

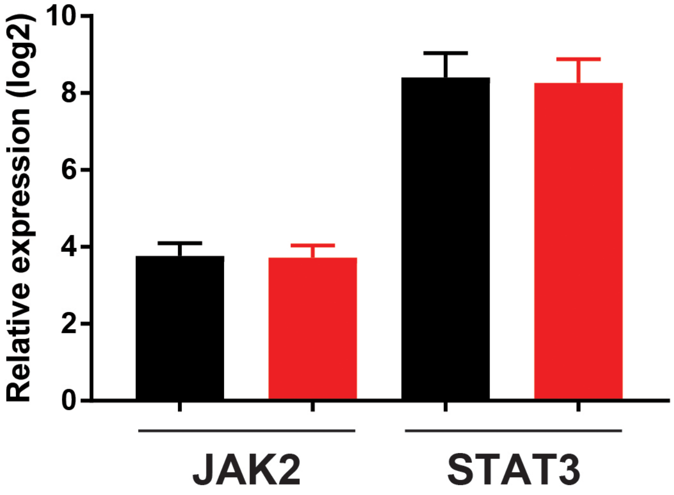

Supplementary Figure 7: Expression of JAK2 and STAT3 in patient cohort. Relative expression of JAK2 and genes STAT3 in ovarian cancer patients expressing high (above median, red) versus low (below median, black) levels of both *LEP* and *LEPR*.
